# A Common Live-Attenuated Avian Herpesvirus Vaccine Expresses a Very Potent Oncogene

**DOI:** 10.1128/mSphere.00658-19

**Published:** 2019-10-09

**Authors:** Andelé M. Conradie, Luca D. Bertzbach, Nirajan Bhandari, Mark Parcells, Benedikt B. Kaufer

**Affiliations:** a Institut für Virologie, Freie Universität Berlin, Berlin, Germany; b Department of Animal and Food Sciences, University of Delaware, Newark, Delaware, USA; University of Arizona

**Keywords:** vaccine, CVI988/Rispens, Marek’s disease virus (MDV), tumorigenesis, T cell lymphoma, cancer, *meq* gene, basic leucine zipper (bZIP) protein, herpesvirus telomerase RNA (vTR)

## Abstract

Vaccines play a crucial role in the protection of animals and humans from deadly pathogens. The first vaccine that also protected against cancer was developed against the highly oncogenic herpesvirus Marek’s disease virus (MDV). MDV infects chickens and causes severe immunosuppression, neurological signs, and fatal lymphomas, a process that requires the viral oncogene, *meq*. The most frequently used Marek’s disease vaccine is the live-attenuated CVI988/Rispens (CVI) strain, which efficiently protects chickens and prevents tumorigenesis. Intriguingly, CVI expresses at least two isoforms of *meq*; however, it remains unknown to what extent these isoforms contribute to virus attenuation. In this study, we individually examined the contribution of the two CVI*-meq* isoforms to the attenuation of the vaccine. We inserted the respective isoforms into a very virulent MDV (strain RB-1B), thereby replacing its original *meq* gene. Surprisingly, we could demonstrate that the longer isoform of *meq* strongly enhanced virus-induced pathogenesis and tumorigenesis, indicating that other mutations in the CVI genome contribute to virus attenuation. On the contrary, the shorter isoform completely abrogated pathogenesis, demonstrating that changes in the *meq* gene can indeed play a key role in virus attenuation. Taken together, our study provides important evidence on attenuation of one of the most frequently used veterinary vaccines worldwide.

**IMPORTANCE** Marek’s disease virus (MDV) is one of several oncogenic herpesviruses and causes fatal lymphomas in chickens. The current “gold standard” vaccine is the live-attenuated MDV strain CVI988/Rispens (CVI), which is widely used and efficiently prevents tumor formation. Intriguingly, CVI expresses two predominant isoforms of the major MDV oncogene *meq*: one variant with a regular size of *meq* (*Smeq*) and one long isoform (*Lmeq*) harboring an insertion of 180 bp in the transactivation domain. In our study, we could break the long-standing assumption that the *Lmeq* isoform is an indicator for virus attenuation. Using recombinant viruses that express the different CVI-*meq* isoforms, we could demonstrate that both isoforms drastically differ in their abilities to promote pathogenesis and tumor formation in infected chickens.

## Abstract (precis)

Marek’s disease virus (MDV) is one of several oncogenic herpesviruses and causes fatal lymphomas in chickens. The current “gold standard” vaccine is the live-attenuated MDV strain CVI988/Rispens (CVI), which is widely used and efficiently prevents tumor formation. Intriguingly, CVI expresses two predominant isoforms of the major MDV oncogene *meq*: one variant with a regular size of *meq* (*Smeq*) and one long isoform (*Lmeq*) harboring an insertion of 180 bp in the transactivation domain. In our study, we could break the long-standing assumption that the *Lmeq* isoform is an indicator for virus attenuation. Using recombinant viruses that express the different CVI-*meq* isoforms, we could demonstrate that both isoforms drastically differ in their abilities to promote pathogenesis and tumor formation in infected chickens.

## INTRODUCTION

**FIG 1:**
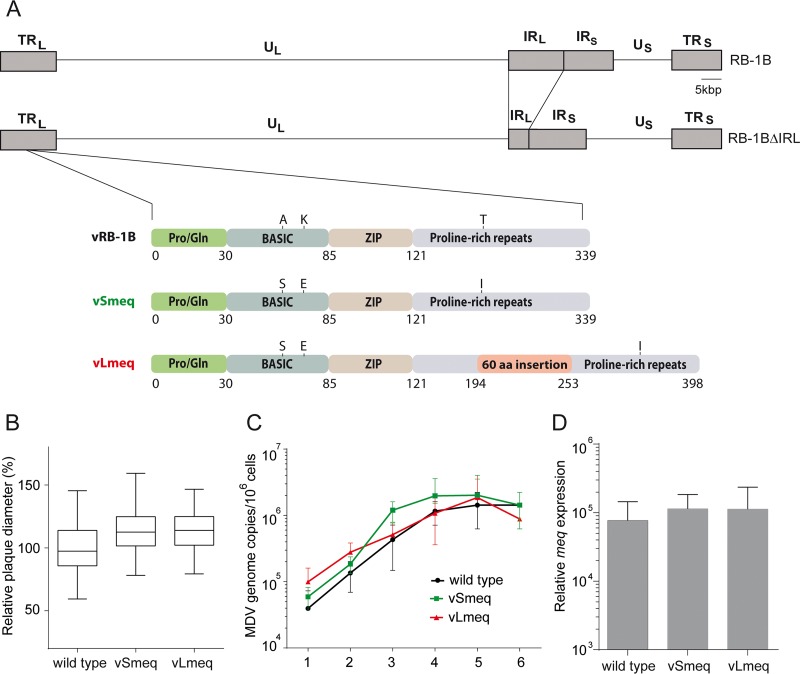
Construction and *in vitro* characterization of recombinant viruses. (A) Schematic representation of the MDV RB-1B genome with a focus on the different *meq* genes, including mutations in the basic domain and proline-rich repeats. (B) Virus spread was assessed by plaque size assays (*n* = 150) and replication by (C) multistep growth kinetics 1 to 6 days postinfection. Spread and replication of indicated recombinant viruses were not statistically different (*P* > 0.05, one-way ANOVA). (D) *meq* expression levels in infected chicken embryo cells relative to GAPDH were not statistically different (*P* > 0.05, Kruskal-Wallis test). Data are shown as the means from a minimum of three independent experiments with standard deviation (SD [shown by error bars]).

Marek’s disease virus (MDV) is a lymphotropic alphaherpesvirus that infects chickens and causes 1 to 2 billion dollar losses worldwide annually (1). MDV causes a variety of clinical symptoms, including immunosuppression, ataxia, chronic wasting, and formation of T cell lymphoma in various visceral organs (2). MDV vaccines are widely used to protect chickens from this deadly disease and were the first vaccines that prevented cancer, long before this approach was applied to human medicine (3, 4). The current “gold standard” vaccine is the live-attenuated MDV strain, CVI988/Rispens (CVI), which efficiently protects chickens against very virulent field strains (5, 6). Intriguingly, commercial vaccine stocks express two predominant isoforms of the major MDV oncogene *meq* (7). The Meq protein is a basic leucine zipper (bZIP) protein that is essential for tumorigenesis, represses apoptosis, dysregulates the cell cycle, and modulates cellular and viral gene expression (8–10). One of the cellular targets is c-*myc*, which influences the expression of MDV-encoded viral telomerase RNA (vTR) (11), a noncoding RNA that plays an important role in tumorigenesis (12). One of the two CVI-carried *meq* genes has the same size as its counterparts in virulent MDV strains, but harbors several point mutations (*Smeq*) (Fig. 1A) (13). The other isoform is identical to *Smeq* except for an in-frame insertion (*Lmeq*) of 180 bp (60 amino acids) in the carboxy-terminal transactivation domain (14, 15). The insertion consists of proline-rich repeats (PRR) that likely arose from a domain duplication (16). It has been shown that these two CVI-*meq*s are weak transactivators of viral gene expression, which could contribute to the nononcogenic phenotype of the CVI virus in chickens (7). To determine the role of the *meq* isoforms expressed in the CVI vaccine, we replaced the *meq* gene in the very virulent MDV strain RB-1B with either the *Smeq* (vSmeq) or *Lmeq* (vLmeq) isoform. Intriguingly, we found that viruses with these vaccine-derived *meq* isoforms strikingly differ in pathogenesis and oncogenesis in infected chickens.

## RESULTS

### Generation of recombinant viruses.

To examine the contribution of the two *meq* isoforms in the attenuation of the CVI vaccine, we generated recombinant viruses that harbor either the *Smeq* or *Lmeq* isoform. CVI-*meq* isoforms were individually inserted into the very virulent RB-1B MDV strain instead of its native *meq* gene (Fig. 1A). We confirmed the resulting clones with PCR, restriction fragment length polymorphism (RFLP), Sanger sequencing, and Illumina MiSeq whole-genome sequencing with an ∼1,000-fold coverage to confirm the integrity and the sequence of the entire virus genome.

### *In vitro* characterization of recombinant viruses.

To determine if insertion of the *meq* isoforms affects virus replication, we performed plaque size assays and examined multistep growth kinetics. We could demonstrate that the recombinant viruses efficiently replicate similar to the parental (wild-type) virus (Fig. 1B and C), indicating that the insertion of the CVI-*meq* isoforms in a very virulent RB-1B strain does not affect virus replication *in vitro*. To ensure that both CVI-*meq* isoforms are efficiently expressed, we quantified the expression levels of *meq* in virus-infected cells by quantitative reverse transcription-PCR (RT-qPCR). We could demonstrate that the *meq* gene expression of vSmeq or vLmeq was comparable to the *meq* expression in the wild-type virus (Fig. 1D).

### Replication of recombinant viruses *in vivo*.

**FIG 2:**
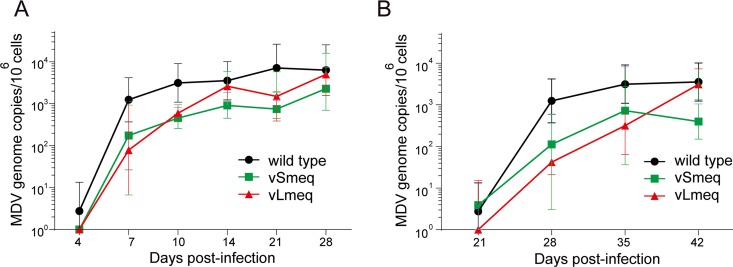
Replication of recombinant viruses *in vivo*. MDV genome copies were detected in blood samples of (A) chickens infected with indicated viruses as well as in (B) contact chickens infected via the natural route by qPCR. Genome copy numbers were not statistically different (*P* > 0.05, Kruskal-Wallis test).

To assess if the CVI-*meq* isoforms affect virus replication, pathogenesis, and/or tumor formation *in vivo*, we infected 1-day-old Valo specific-pathogen-free (SPF) chickens subcutaneously with 4,000 PFU of wild-type virus, vSmeq, or vLmeq. The viral load in the blood was assessed by qPCR and revealed that the recombinant viruses replicated efficiently in infected animals (Fig. 2A), indicating that the CVI-*meq* isoforms do not affect virus replication *in vivo*. Moreover, all viruses efficiently spread to cohoused contact chickens, confirming that the insertion of *Smeq* and *Lmeq* did not significantly influence virus transmission to naive contact chickens (Fig. 2B).

### Pathogenesis and tumorigenesis of recombinant viruses *in vivo*.

**FIG 3:**
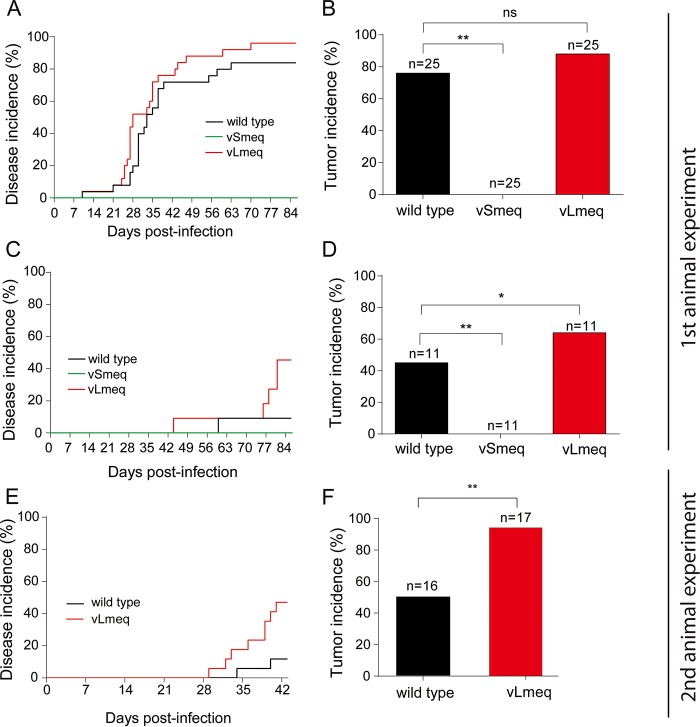
*In vivo* characterization of recombinant viruses. Kaplan-Meier analyses of Marek’s disease incidence in chickens infected with indicated recombinant viruses (A and E) and naive chickens infected via the respiratory route (C) in two independent animal experiments. Statistical analyses using the log-rank test revealed a significant difference between vLmeq and vSmeq in panels A (*P* = 0.0001) and C (*P* = 0.0142). A significant difference between vLmeq and wild type was observed in panels C (*P* = 0.0142) and E (*P* = 0.02). Tumor incidences are shown as percentage per group in infected chickens (B and F) and in naive contact chickens (D). Asterisks indicate significant differences (*, *P* < 0.05, and **, *P* < 0.0125, Fisher’s exact test). ns, not significant.

We monitored the animals for clinical symptoms and tumor development over the course of the experiment. The recombinant virus harboring the *Smeq* isoform did not cause any disease (Fig. 3A), indicating that the small number of amino acid changes in *meq* can indeed attenuate the virus. Surprisingly, an increase in disease was observed in animals infected with vLmeq (Fig. 3A), revealing that the 180-bp insertion in *Lmeq* enhances the potency of the *meq* oncogene. Almost all the chickens infected with vLmeq succumbed to disease (96%), while a lower incidence was observed for wild-type virus (84%). Consistently, contact animals infected via the natural route with vLmeq had a significantly higher disease incidence (45%) compared to the wild-type control (9%). No pathogenicity was observed in the vSmeq contact animals (Fig. 3C). We confirmed the increased disease incidence caused by vLmeq in a second independent animal experiment (Fig. 3E).

**FIG 4:**
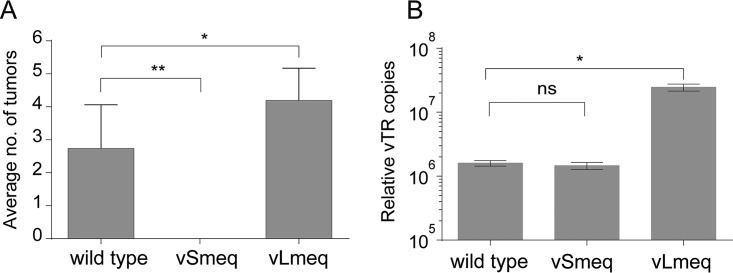
Analysis of tumor dissemination and vTR expression of recombinant viruses. (A) Mean number of organs with gross tumors per animal in the indicated groups (1st animal experiment). Significant differences are indicated by asterisks (*, *P* < 0.05, and **, *P* < 0.0125, Fisher’s exact test). (B) Mean genome copies of vTR for the indicated viruses are shown relative to the cellular GAPDH (*, *P* < 0.05, Kruskal-Wallis test; *n* = 3). ns, not significant.

In addition, we quantified the number of animals that developed macroscopic tumors. Remarkably, the virus harboring the *Lmeq* showed the highest tumor incidence (88%) compared to wild-type virus (76%) (Fig. 3B), while no tumors were present in the vSmeq group (Fig. 3B). In line with this, an increase in the tumor incidence was also observed in vLmeq contact chickens (64%) compared to the wild-type virus group (45%) (Fig. 3D). In the case of vSmeq, none of the contact animals developed tumors, confirming the data from the experimentally infected animals (Fig. 3D). This increased tumor incidence was confirmed by an independent animal experiment (Fig. 3F). Intriguingly, tumor dissemination was also enhanced in vLmeq-infected chickens as more organs harbored tumor lesions per animal (Fig. 4A), highlighting the high oncogenic potential of the *Lmeq* isoform.

### Role of CVI-*meq* isoforms in vTR expression.

To provide a possible mechanistic explanation for the increased oncogenic potential of the *Lmeq* isoform, we examined the expression of vTR in cells infected with the wild type, vSmeq, and vLmeq using RT-qPCR. Remarkably, the *Lmeq* isoform significantly upregulated vTR by 12-fold compared to the already highly expressed RB-1B-carried *meq* and *Smeq*, suggesting that the 180-bp insertion in the transactivation domain strongly influences vTR expression and in turn transformation efficiency in chickens (Fig. 4B). The increase in vTR copies thereby provides a reasonable explanation for the increased tumor-promoting activity of the *Lmeq* isoform in chickens.

## DISCUSSION

The CVI vaccine is the current “gold standard” vaccine against MDV and efficiently protects against very virulent MDV strains. In previous studies, we and others observed that at least two *meq* isoforms are expressed in vaccine stocks (7, 17, 18). The two predominant *meq* isoforms expressed from the vaccine are *Smeq* and *Lmeq* and were considered weak oncogenes due to that fact that the CVI vaccine does not induce tumors. These two *meq* isoforms differ by an insertion of 180 bp in the transactivation domain (*Lmeq*) compared to the *Smeq*. Both isoforms are only expressed in the CVI vaccine and have not been detected in other MDV strains, such as RB-1B and MD5 (17). In this study, we individually delineated the contribution to attenuation and the oncogenic potential of *Smeq* and *Lmeq*.

First, we performed growth kinetics and plaque assays to determine if the insertion of *Smeq* and *Lmeq* affects growth properties, as the oncogene is also expressed during lytic replication. Our results show that replacing the original *meq* of the very virulent RB-1B strain with the CVI-*meq* isoforms does not significantly influence its growth properties *in vitro* (Fig. 1B and C). Furthermore, we could demonstrate that the *meq* gene expression of vSmeq or vLmeq was comparable to its counterpart in the wild-type virus (Fig. 1D), confirming that the observed effects in this study are not due to differences in the *meq* expression levels.

Next, we characterized the recombinant viruses *in vivo* and found that insertion of *Smeq* completely abrogated MDV pathogenesis and oncogenesis. The inserted *Smeq* only differs by three amino acid changes compared to the wild-type *meq* from the very virulent RB-1B MDV strain. It is intriguing that this small number of amino acid changes in *meq* could completely attenuate the very virulent RB-1B strain. In contrast, pathogenesis and oncogenesis were severely enhanced upon insertion of *Lmeq*. It is remarkable that an insertion of only 180 bp in the PRR of *Smeq* drastically enhanced disease incidence (Fig. 3A) and tumorigenesis (Fig. 3B and 4A) in infected animals. The same trend was observed in contact chickens that were infected via a natural route. All recombinant viruses were able to spread efficiently to contact chickens (Fig. 2B). We observed a slight delay with vLmeq; however, this was not statistically significant. Only wild-type and vLmeq viruses were able to cause disease (Fig. 3C) and tumors (Fig. 3D) in the contact chickens. An independent animal experiment using a different chicken line confirmed the markedly elevated disease incidence (Fig. 3E) and the high oncogenic potential of vLmeq (Fig. 3F).

To explain the *in vivo* data, we focused on a viral gene that (i) plays a role in transformation and (ii) is regulated by the Meq protein. It has previously been shown that the Meq protein modulates the expression levels of vTR, which plays a crucial role in MDV-induced lymphomagenesis and tumor dissemination via cellular c-*myc* (11, 12, 19). We quantified the expression levels of vTR in cells infected with the respective viruses and could show that expression of the *Lmeq* isoform significantly upregulates vTR compared to the wild-type *meq* and *Smeq.* This suggests that the 180-bp insertion in the transactivation domain of *Lmeq* strongly influences vTR expression via c-*myc* (Fig. 4B), and could therefore explain the increased tumorigenesis observed in chickens infected with vLmeq in both animal experiments.

In addition, our data on *Lmeq* suggest that other mutations in the CVI genome contribute to attenuation of the vaccine, resulting in a fully attenuated virus despite the presence of this potent oncogene. Strikingly, CVI harbors a number of mutations/indels and amino acid changes compared to virulent strains as published previously (16); however, whether these changes have an effect on oncogenesis remains elusive. An alternative explanation for the apathogenic nature of CVI would be that the oncogenic potential of *Lmeq* is masked by heterodimerization with the *Smeq* isoform. It remains unknown if *Smeq* and *Lmeq* can repress each other, an aspect that will be addressed in future studies. In previous studies, coexpression of CVI *Smeq* or *Lmeq* with the oncogene of the MD5 strain resulted in a suppression of the *meq* promoter (7, 20). However, this suppressive effect was not observed in cells infected with our recombinant viruses expressing *Smeq* and *Lmeq* individually (Fig. 1D).

In summary, we assessed the contribution of the CVI-*meq* isoforms to the attenuation of the vaccine strain. Our study revealed that the two CVI-*meq* isoforms allow efficient virus replication; however, they vastly differ in their tumor-promoting properties. Strikingly, the *Lmeq* isoform enhances MDV pathogenesis and oncogenesis of a very virulent MDV strain, while insertion of the *Smeq* isoform completely abrogated MDV pathogenesis. Our results from the *Lmeq* isoform break with the long-standing assumption that it is a marker for attenuation (21–23) and demonstrate that other mutations in the CVI genome contribute to its attenuation.

## MATERIALS AND METHODS

### Cells.

Primary chicken embryo cells (CECs) were prepared from 11-day-old specific-pathogen-free (SPF) chicken embryos (Valo BioMedia, Germany) as described previously (24). Cells were cultured in Eagle’s minimal essential medium (MEM) supplemented with 10% bovine serum and antibiotics (100 U/ml penicillin and 100 μg/ml streptomycin) at 37°C in a humidified atmosphere containing 5% CO_2_.

### Generation of recombinant viruses.

We generated recombinant viruses each harboring either *Smeq* or *Lmeq* (GenBank accession no. AY243333 and AY243338) derived from the commercial CVI988/Rispens vaccine strain (5). *Smeq* and *Lmeq* were inserted into a bacterial artificial chromosome (BAC) of the very virulent MDV strain RB-1B, which lacks most of the internal repeat long region (IRL; pRB-1BΔIRL), which is rapidly restored upon virus reconstitution (25). Therefore, only one copy of the *meq* region had to be manipulated by two-step Red-mediated mutagenesis as described previously (26, 27), while the resulting recombinant virus contained the *meq* substitution in both loci as confirmed by PCR (25). First, we deleted the *meq* gene and then introduced either *Smeq* (vSmeq) or *Lmeq* (vLmeq). We confirmed the BAC clones by RFLP, PCR, and Sanger and Illumina MiSeq sequencing (Illumina’s v3 chemistry for 600-bp paired‐end sequencing) to verify the integrity and the sequence of the entire virus genome. The primers used for mutagenesis and sequencing are listed in Table 1. All viruses were reconstituted and propagated on CECs, and stocks were prepared as described previously (25, 28).

**TABLE 1:** Primers and probes used for construction of recombinant viruses, DNA sequencing, and qPCR

Construct	Primer or probe^*a*^	Sequence (5′→3′)^*b*^
*meq* kana_in (transfer construct)	for	AATTCGAGATCTAAGGACTGAGTGCACGTCCCTGTAGGGATAACAGGGTAATCGATTT
	rev	GTCCTTAGATCTCGAATTTCCTTACGTAGGGCCAGTGTTACAACCAATTAACC
Δ*meq* (deletion of RB-1B *meq*)	for	CAGGGTCTCCCGTCACCTGGAAACCACCAGACCGTAGACTGGGGGGACGGATCGTCAGCGGTAGGGATAACAGGGTAATCGATTT
	rev	GGGCGCTATGCCCTACAGTCCCGCTGACGATCCGTCCCCCCAGTCTACGGTCTGGTGGGCCAGTGTTACAACCAATTAACC
IRL restoration (sequencing)	for	CGAACGGAATGTACAACAGCTTGC
	rev	GATAAGACACTTTCCCACTCATAC
MDV_*meq* (insertion of CVI-*meqs*)	for	ATGTCTCAGGAGCCAGAGCC
	rev	GGGTCTCCCGTCACCTGG
ICP4 (qPCR)	for	CGTGTTTTCCGGCATGTG
	rev	TCCCATACCAATCCTCATCCA
	Probe	FAM-CCCCCACCAGGTGCAGGCA-TAM
iNOS (qPCR)	for	GAGTGGTTTAAGGAGTTGGATCTGA
	rev	TTCCAGACCTCCCACCTCAA
	Probe	FAM- CTCTGCCTGCTGTTGCCAACATGC-TAM
*meq* (RT-qPCR	for	TTGTCATGAGCCAGTTTGCCCTAT
	rev	AGGGAGGTGGAGGAGTGCAAAT
	Probe	GGTGACCCTTGGACTGCTTACCATGC
vTR (RT-qPCR)	for	CCTAATCGGAGGTATTGATGGTACTG
	rev	CCCTAGCCCGCTGAAAGTC
	Probe	FAM-CCCTCCGCCCGCTGTTTACTCG-TAM
GAPDH (RT-qPCR)	for	GAAGCTTACTGGAATGGCTTTCC
	rev	GGCAGGTCAGGTGAACAACA
	Probe	FAM-TGTGCCAACCCCCAAT-TAM

a for, forward primer; rev, reverse primer.

b FAM, 6-carboxyfluorescein; TAM, TAMRA.

### Plaque size assays and multistep growth kinetics.

The spread and replication of the recombinant viruses were first analyzed by plaque size assays as described previously (29). Briefly, 1 million CECs were infected with 100 PFU of the recombinant viruses and cells were fixed at 6 days postinfection (dpi). Images of randomly selected plaques (*n* = 50) were taken, and plaque areas were determined using Image J software (NIH).

Plaque size data were confirmed by qPCR-based multistep growth kinetics as described previously (29). Briefly, one million CECs were infected with 100 PFU of the recombinant viruses, and virus replication was assessed by qPCR over 6 days of infection. Primers and probes specific for the MDV-infected cell protein 4 (ICP4) and chicken inducible nitric oxide synthase (iNOS) are shown in Table 1. Virus genome copies were normalized against the chicken iNOS gene as published previously (30).

### RT-qPCR.

To ensure that the CVI-*meq* isoforms are expressed comparable to its counterpart in wild-type RB-1B, we quantified the expression levels of the different *meqs* using RT-qPCR as previously described (31). Briefly, total RNA was extracted from virus-infected CECs using the RNeasy Plus minikit (Qiagen) according to the manufacturer’s instructions. The samples were treated with DNase I (Promega), and cDNA was generated using the High-Capacity cDNA reverse transcription kit (Applied Biosystems). *meq* expression levels were normalized to the expression levels of cellular GAPDH (glyceraldehyde-3-phosphate dehydrogenase). We also used this approach to examine the expression of vTR in cells infected with the wild type and vSmeq and vLmeq strains by RT-qPCR. The vTR expression levels were normalized to the expression levels of cellular GAPDH (32). The primers and probes used for qRT-PCR are shown in Table 1.

### *In vivo* characterization of recombinant viruses.

The replication properties, pathogenesis, and tumorigenesis of the recombinant viruses were assessed in specific-pathogen free (SPF) chickens as described previously (31). In the first animal experiment, 1-day-old Valo SPF chickens (Valo BioMedia) were randomly distributed into three groups. The chickens were infected subcutaneously with 4,000 PFU of the wild type (*n* = 25), vSmeq (*n* = 25), and vLmeq (*n* = 25). Each group was cohoused with 11 noninfected contact animals to assess the natural transmission of the respective virus from experimentally infected birds. The animal experiment was approved by the Landesamt für Gesundheit und Soziales in Berlin, Germany (LAGeSo; approval no. G0294-17) and was conducted according to relevant national and international guidelines for humane use of animals. Animals were monitored daily for clinical symptoms throughout the 86-day experiment.

The phenotype of the vLmeq was confirmed in a second, independent animal experiment. White leghorn chickens (Sunrise Farms, Inc., Catskill, NY) were inoculated with 1,000 PFU of either the wild type (*n* = 16) or vLmeq (*n* = 17). This animal experiment was approved by the Agricultural Animal Care and Use Committee (AACUC; approval no. [22] 05-23-13b-R). Animals were monitored for clinical symptoms throughout the 43-day experiment.

To eliminate bias, the examining veterinarian had no knowledge of the viruses in the different groups. All chickens were humanely euthanized and examined for gross tumor lesions if symptoms appeared or upon termination of the experiment. DNA was isolated from spleens and tumors to confirm the sequence of the respective *meq* gene.

### Quantification of MDV genome copies in blood samples.

The virus load in the blood of infected animals was analyzed at 4, 7, 10, 14, 21, and 28 dpi and for contact animals at days 21, 28, 35, and 42 by qPCR as described previously (33). DNA was isolated from whole-blood samples of infected and contact chickens using the E-Z96 blood DNA kit (OMEGA Biotek, USA) according to the manufacturer’s instructions. We determined MDV genome copy numbers by qPCR using primers and probes specific for the MDV ICP4 as described above.

### Statistical analysis.

Statistical analysis was performed using Graph-Pad Prism v7 and the SPSS software (SPSS, Inc.). Analysis for plaque size assays and growth kinetics included one-way analysis of variance (ANOVA). Fisher’s exact test and Kaplan-Meier survival analysis along with the log-rank test (Mantel-Cox test) were used for analyses of the animal experiment data with Bonferroni correction on multiple comparisons. Differences were considered significant if *P* was <0.0125.
